# Mechanistic Insight in Permeability through Different Membranes in the Presence of Pharmaceutical Excipients: A Case of Model Hydrophobic Carbamazepine

**DOI:** 10.3390/pharmaceutics16020184

**Published:** 2024-01-28

**Authors:** Tatyana Volkova, Olga Simonova, German Perlovich

**Affiliations:** G.A. Krestov Institute of Solution Chemistry RAS, 153045 Ivanovo, Russia; ors@isc-ras.ru (O.S.); glp@isc-ras.ru (G.P.)

**Keywords:** permeability, cellulose membrane, polydimethylsiloxane–polycarbonate membrane, micelle/water partition coefficient, association constant

## Abstract

The present study reports the effects of two pharmaceutical excipients of differing natures—non-ionic surfactant pluronic F127 (F127) and anionic sulfobutylether-β-cyclodextrin (SBE-β-CD)—on the permeation of the model compound, carbamazepine (CBZ). The permeability coefficients of CBZ at three concentrations of the excipients were measured through two different artificial barriers: hydrophilic cellulose membrane (RC) and lipophilic polydimethylsiloxane–polycarbonate membrane (PDS). The equilibrium solubility of CBZ in F127 and SBE-β-CD solutions was determined. The micellization, complexation, and aggregation tendencies were investigated. Systemically increasing the solubility and the reduction of permeation upon the excipients’ concentration growth was revealed. The quantitative evaluation of the permeability tendencies was carried out using a *P_ratio_* parameter, a quasi-equilibrium mathematical mass transport model, and a correction of permeability coefficients for the free drug concentration (“true” permeability values). The results revealed the mutual influence of the excipient properties and the membrane nature on the permeability variations.

## 1. Introduction

Membrane permeability along with the solubility in the biological fluids and stability in the gastrointestinal environment serves as one of the major determinants of bioavailability [1]. Permeability is crucial for therapeutic response, dose selection, and, in many cases, is responsible for a degree of undesirable effects [2]. Its significance arises for the peroral root of administration—the most convenient and widely applied approach in which a drug substance may need to pass through several membranes [3].

Interestingly, nearly 40% of the drugs on the market and 90% on the stage of design are poorly soluble in water, can be classified into class II and class IV of the Biopharmaceutics Classification System (BCS) [4], and continue to be a problematic yet important for treatment of a wide range of diseases. Furthermore, approximately 35% of the marketed drugs and 30% of the drug compounds under development at the moment suffer from permeability-related difficulties [5].

A large number of approaches aimed at the solubility improvement via the development of solubility-enhancing formulations, such as the addition of co-solvents, biopolymers [6], surface active substances, the forming of micelles in the solution [7], complexation with cyclodextrins [8], pharmaceutical salts and cocrystals [9], liposomes [10], and even protein-based compositions [4], have been developed.

At the same time, a fact of reducing the permeability upon the solubility growth in the presence of solubilizing agents has been reported in many studies, for example, by Dahan, Miller, and co-workers [11,12], and also in some of the studies of our research team [13]. It is clear that the optimal ratio between solubility and permeability is an indispensable condition of advantageous drug formulation.

In order to reach an adequate permeability, it seems important to shed light on the factors that govern it in the presence of pharmaceutical additives that serve as solubilizers, stabilizers, wetting agents, etc. A large number of studies discuss the possible reasons of permeability modulations, such as variations of the diffusion coefficient and the hydrodynamic radius of the permeated molecule, the viscosity of the donor medium, and altering the barrier properties through drug–membrane and membrane–medium interactions [14,15]. A generalized method for the estimation of the permeability of passively transported drugs under solubilizing experimental conditions was proposed by Katneny et al. [16] and was tested on several poorly soluble drugs. This approach enables the evaluation of “true” permeability via the simple extrapolation of measured permeability coefficients which are obtained at a minimum of three surfactant concentrations above the CMC in the case of micellar solutions. The mechanistic investigation into the increase in apparent solubility, induced by the liposomalization or micellization of the poorly soluble drug hydrocortisone and the possibility of the permeability enhancement, was demonstrated by di Cagno and Luppi [17]. Interestingly, the authors estimated a direct proportion between the concentration of the molecularly dissolved drug and the respective fluxes, but no correlation of the apparent solubilities with fluxes was found. In the study of the permeability of three chemically diverse compounds, atenolol, ketoprofen, and hydrocortisone with cyclodextrins and liposomes, Tzanova et al. [18] revealed that solubilizing with cyclodextrins may or may not vary drug permeation. In turn, in the case of liposomal systems, drug permeability can be directly correlated to the free drug concentration. The authors [19] managed to discriminate precisely between the amount of drugs bound to nanocarriers or those that were freely dissolved at any time point using UV–Vis localized spectroscopy and mathematical modeling. The increasing number of the articles devoted to identifying the role of the free drug concentration indicates the importance of this issue for assessing the permeability in the solubilizing conditions. 

The theoretic basis of permeability reduction in the presence of cyclodextrins, surfactants, polymers, and other additives can be attributed to the reduction in the thermodynamic activity of the compound [16]. This phenomenon is responsible for the variations of the free drug concentration. As it was demonstrated [20], only molecularly dissolved drugs—free molecules surrounded by a solvation shell (not solubilized)—are capable of penetrating the biological barriers. In this respect, in most cases, just the concentration of molecularly dissolved drugs seems to be taken into account when concerning the permeability of the drug in the context of solubility-enabling formulations [21]. However, as it was shown in the recent study by Tzanova et al. [18], at high concentrations, the complex can also penetrate through the barrier. From all the premises, it seems reasonable to make additional investigations aimed at the elucidation of all the factors concerning the permeability of drugs in pharmaceutical formulations in order to make correlations regarding absorption rate.

For a long time, the permeation of drugs in vitro has been assessed with different artificial barriers. The cell-free permeation models were shown to be more robust against pharmaceutical excipients, and their field of use has been expanded to transdermal, buccal, and even blood–brain barrier permeation [22]. Among others, the hydrophilic membranes based on the regenerated cellulose have been intensively used for the purposes of the comparison of the permeability between various drug compositions. As it was reported [22], a cellulose membrane with a molecular cutoff weight of 12–14 kDa is regularly performed to estimate the accessible drug fraction. Being applicable for diffusion rate evaluations, this barrier is hydrophilic, capable of water permeation, and cannot simulate the lipophilic layer of cell membranes. In their turn, lipophilic barriers, such as polydimethylsiloxane–polycarbonate membranes [23] and phospholipid-based PermeaPad barriers [24], are applied not only for the sake of comparison between different formulations, but also to simulate the permeability by transcutaneous and intestinal/buccal delivery, respectively. As has been identified within the literature, the PDS membrane has a heterophase and heteropolar structure that is similar to the human skin epidermis [23].

In the present study, we used carbamazepine (CBZ) as an ideal model compound that has been thoroughly investigated by many authors. CBZ is a dibenzazepine derivative which has been widely applied in the treatment of trigeminal neuralgia, manic-depressive illness, and epilepsy [25]. As it was reported [26], in an aqueous solution, CBZ rapidly transforms into CBZ dihydrate. The selection of CBZ was caused by its poor solubility that makes it a suitable substrate for cyclodextrins and surfactants, such as, for example, hydrocortisone and ketoprofen in the study of Tzanova et al. [18]. Sulfobutylether-β-cyclodextrin and pluronic F127 were chosen as the excipients. Sulfobutylether-β-cyclodextrin—a safe solubilizer and stabilizer—was taken as a superior hydrophilic cyclodextrin, useful as a formulation component [27,28]. Pluronics have long been accepted for potent drug delivery due to their ability to form micelles [29]. Pluronic F127 of a high molecular weight (M_w_ = 12,600 Da) was shown to display higher drug loading efficiency and prolonged drug release when compared to pluronics with lower M_w,_ by means of enhanced hydrophobic interactions [30,31].

**Figure 1:**
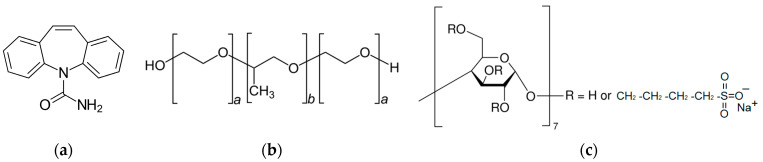
Structures of the studied objects: carbamazepine (**a**), pluronic F127 (**b**), sulfobutylether-β-CD (**c**).

The aim of this work was to evaluate the influence of different contributions to the in vitro permeability of a model drug—carbamazepine (CBZ) (Figure 1a) in the presence of pharmaceutical excipients, pluronic F127 (F127) (Figure 1b) and sulfobutylether-β-cyclodextrin (SBE-β-CD) (Figure 1c). To this end, the permeability experiments were carried out in the presence of different concentrations of the excipients. The impact of the membrane type on the permeation regularities was disclosed with the help of two membranes: hydrophilic cellulose membrane (RC) and lipophilic polydimethylsiloxane–polycarbonate membrane (PDS). The obtained parameters were evaluated quantitatively through several mechanistic models.

## 2. Materials and Methods

### 2.1. Materials

Carbamazepin (CBZ), C_15_H_12_N_2_O, purity 98%, and pluronic F127 (M_w_ = 12,600 Da) were purchased from Acros Organics, Thermo Fisher Scientific, Geel, Belgium. Sulfobutylether-β-CD, purity 99%, was received from BLDpharm (https://www.bld-pharm.com/ (accessed on 2 April 2019)). Potassium dihydrogen phosphate (purity ≥99%) and sodium hydroxide (purity ≥98%) were supplied by Merk.

The phosphate buffer pH 6.8 was made as follows: 27.22 g of KH_2_PO_4_ was dissolved in 1 L of water (Solution 1); 2 g of NaOH was added to 250 mL of H_2_O (Solution 2). Then, 250 mL of Solution 1 and 112 mL of Solution 2 were mixed together and diluted with bi-distilled water to 1 L.

All the reagents and solvents were used as received. A FG2-Kit pH meter (Mettler Toledo, Switzerland) standardized with pH 4.00 and 7.00 solutions was used to check the pH of the prepared buffers.

### 2.2. Determination of CBZ Equillibrium Solubility in F127 and SBE-β-CD Solutions

The solubility of CBZ was measured in pure buffer pH 6.8 at 37 °C by the standard shake-flask method [32], with the additions of 1.33, 1.83, 2.30 mmol·L^−1^ and 6.89, 13.78, 20.67 mmol·L^−1^ of F127 and SBE-β-CD, respectively, which corresponded to 1%, 2%, and 3% (*w*/*v*). The experimental temperature of 37 °C corresponded to the standard temperature of a healthy human. The excess amounts of CBZ were placed into screw capped vials containing the buffer solutions, both with and without the excipients. The vials were continuously shaken in an air thermostat to achieve the equilibrium. After this, the suspensions were settled during no less than 6 h to avoid supersaturation [33]. The clear solutions were obtained through filtration (PTFE syringe filter 0.22 μm), and the concentration of CBZ was determined via HPLC using the calibration curves with 2–4% accuracy. The experimental results are presented as an average of at least three replicated experiments. 

### 2.3. HPLC Analysis

The samples of the solutions were analyzed via HPLC using the Shimadzu Prominence model LC-20 AD, equipped with a PDA detector and a C-18 column Luna^®^ (150 mm × 4.6 mm i.d., 5 μm particle size, and 100 Å pore size) (Albert-Hahn-Str. 6-10, Duisburg, Germany). The column temperature was 40 °C. Eluent acetonitrile:water at a ratio of 40:60 *v*/*v* was used. An isocratic regime at a flow rate of 1 mL·min^−1^ was applied. The injection volume was 20 μL. CBZ was detected (UV) at 284 nm with a retention time of 4.95 min.

### 2.4. Determination of CMC of F127 and CAC of SBE-β-CD in Buffer Solution pH 6.8

The critical micelle concentration (CMC) of F127 and the critical aggregation concentration (CAC) of SBE-β-CD in the absence and in the presence of CBZ were determined via measuring the refraction index with the help of a laboratory differential refractometer (IRF-454 B2M G34.15.051; Produced: OAO “Thermoinstrument”, Klin, Russia; https://www.pribor.com, (accessed on 16 June 2023)) according to the literature [34]. The plot of the refractive index dependence on the F127 concentration represents the premicellar and postmicellar regions. The intersection point of these two dependences is considered as *CMC*. In the case of SBE-β-CD, the *CAC* was determined in a similar way.

### 2.5. Estimation of the CBZ/F127 Association Constant, and the Stability Constant of CBZ/SBE-β-CD Complex 

The CBZ/F127 association constant (*K_a_*) and the stability constant of CBZ/SBE-β-CD complex (*K_c_*) were determined, taking into account that the lipophilic CBZ molecule exists in equilibrium between the free (*C_free_*) and the micellar (*C_micellar_*) (in the case of F127) or complexed (*C_complex_*) (in the case of SBE-β-CD) forms, according to the following equilibrium:

(1) $$C_{total}=C_{free}+C_{micellar}$$

(2) $$C_{total}=C_{free}+C_{complex}$$

where *C_total_* is the total CBZ concentration, *C_free_* is the free CBZ concentration, *C_micellar_* and *C_complex_* are the concentration of CBZ in the micellar form and in the form of the CBZ/SBE-β-CD complex. The equilibrium between free and micellar or complexed CBZ can be expressed by the association or stability constant, respectively:

(3) $$K_{a}=\frac{C_{micellar}}{C_{free}\cdot C_{F127}}$$

(4) $$K_{c}=\frac{C_{complex}}{C_{free}\cdot C_{SBE\text{-}\beta \text{-}CD}}$$

where *C_free_* is the free CBZ concentration, *C_micellar_* and *C_complex_* are the concentrations of CBZ in the micellar form and in the form of the CBZ/SBE-β-CD complex, *C*_*F*127_ and *C_HP-β-CD_* are the concentrations of F127 and SBE-β-CD, respectively. In the case of F127, *C_free_* is equal to the equilibrium CBZ solubility ($S_{2}^{0}(CBZ)$) in the saturated solution of pure buffer without F127. A plot of *C_total_* of CBZ against *C*_*F*127_ was used to determine the association constant. To this end, in order to assess the concentration of F127 in the micellar form, the difference between the total F127 (*C*_*F*127_) concentration and the *CMC* was used. In its turn, the stability constant of the CBZ/SBE-β-CD complex was derived from the slope of the linear dependence of the CBZ equilibrium solubility at different concentrations of SBE-β-CD ($S_{2}(CBZ)$) on the SBE-β-CD concentration (*C_SBE-β-CD_*).

### 2.6. Free CBZ Fraction Estimation

The free CBZ fraction at various F127 and SBE-β-CD concentrations was calculated as follows [16]:

(5) $$f_{free}=\frac{C_{free}}{C_{total}}=\frac{1}{\left(1+K_{a}\cdot (C_{F127}-CMC\right)}\;(\mathrm{case}\;\mathrm{of}\;F127)$$

(6) $$f_{free}=\frac{C_{free}}{C_{total}}=\frac{1}{\left(1+K_{c}\cdot C_{SBE\text{-}\beta \text{-}CD}\right)}\;(\mathrm{case}\;\mathrm{of}\;\mathrm{SBE-\beta -CD})$$

where *f_free_* is the free CBZ fraction; *C_free_* is the free CBZ concentration; *C_total_* is the total CBZ concentration (sum of the free and the micellar); K_a_ is the micellar CBZ/F127 association constant; (*C*_*F*127_ − *CMC*) is the concentration of F127 in the micellar form; *K_c_* is the stability constant of CBZ/SBE-β-CD complex; and *C_SBE-β-CD_* is the concentration of SBE-β-CD.

### 2.7. Solubilizing Capacity Calculation

The solubilizing capacity (χ) equaling to the amount of CBZ solubilized by one mole of F127 is expressed by the equation [35]

(7) $$\chi =\frac{\left(S_{2}(CBZ)-S_{2}^{0}(CBZ)\right)}{\left(C_{F127}-CMC\right)}$$

where *χ* is the solubilizing capacity; $S_{2}(CBZ)$ and $S_{2}^{0}(CBZ)$ are the CBZ total solubility in F127 containing buffer solutions and in pure buffer, respectively; *C*_*F*127_ is the total concentration of F127; *CMC* is the critical micelle concentration of F127; (*C _F127_* − *CMC*)—is the concentration of F127 in the micellar form. The solubilizing capacity was determined from the slope of $\left(S_{2}(CBZ)-S_{2}^{0}(CBZ)\right)$ on $\left(C_{F-127}-CMC\right)$.

### 2.8. Light Scattering Measurements

The light scattering measurements were performed using Zetasizer Nano-ZS (Zetasizer Nano ZS, Malvern Instruments Ltd., Malvern, UK) at a scattering angle of 90°. The light source was a He-Ne gas laser which operated at 633 nm. The samples represented the clear solutions and were prepared without any filtration in order to avoid the precipitation on the filter. Each experiment was repeated at least three times.

### 2.9. Aggregation Number Determination

The aggregation number (*N_agg_*) of F127 micelles in the buffer with a pH of 6.8 in the absence and presence of CBZ was determined by the equation taken from [36]:

(8) $$N_{agg}(F127)=\frac{M_{w}(micelle)}{M_{w}(monomer)}$$

where *M_w_* (micelle) and *M_w_* (monomer) are the molecular weights of F127 micelle and F127 monomer, respectively. To determine the average molecular weight of micelles [37], the static light scattering, and the Debye equation were used:

(9) $$\frac{H\cdot (C_{F127}-CMC)}{R}=\frac{1}{M_{w}(micelle)}+2\cdot A_{2}\cdot (C_{F127}-CMC)$$

where *H* is the optical constant, *R* is the excess Rayleigh ratio at an angle of 90°, *A_2_* is the 2nd virial coefficient, *C*_*F*127_ is the pluronic F127 concentration expressed in g·mL^−1^, and *CMC* is the critical micelle concentration in g·mL^−1^.

### 2.10. CBZ Partition Coefficients Determinations

The micelle/buffer apparent partition coefficient (*K_m/buf_* = *K*_*F*127/*buf*_) of CBZ was determined as a ratio between the drug fractions in the micellar and buffer phases by the equation taken from the literature [38]:

(10) $$S_{2}(CBZ)=S_{2}^{0}(CBZ)\cdot (1+K_{F127/buf}\cdot (C_{F127}-CMC)$$

where *K*_*F*127/*buf*_ is the partition coefficient of CBZ between the F127 micellar and aqueous buffer phases, $S_{2}(CBZ)$ and $S_{2}^{0}(CBZ)$ are the CBZ total solubility in F127 containing buffer solutions and in pure buffer, respectively, and (*C*_*F*127_ − *CMC*) is the concentration of F127 in the micellar form. The *K*_*F*127/*buf*_ value was derived from the slope of the plot of ($S_{2}(CBZ)$/$S_{2}^{0}(CBZ)$) on the (*C*_*F*127_ − *CMC*) linear dependence. Similarly, the cyclodextrin/buffer apparent partition coefficient (*K_SBE-β-CD/buf_*) of CBZ can be evaluated using the equation proposed in [39]:

(11) $$S_{2}(CBZ)=S_{2}^{0}(CBZ)\cdot (1+K_{SBE\text{-}\beta \text{-}CD/buf}\cdot C_{SBE\text{-}\beta \text{-}CD})$$

where *K_SBE-β-CD/buf_* is the partition coefficient of CBZ between SBE-β-CD and aqueous buffer phases and $S_{2}(CBZ)$ and $S_{2}^{0}(CBZ)$ are the CBZ total solubility in SBE-β-CD containing buffer solutions and in pure buffer, respectively. In this case, the value of the molar *K_SBE-β-CD/buf_* was derived as the slope of the linear $S_{2}(CBZ)$/$S_{2}^{0}(CBZ)$ on the *C_SBE-β-CD_* dependence. Additionally, for the sake of the reliable comparison of the partition within the micellar and cyclodextrin systems, the concentrations of the excipient (F127 or SBE-β-CD) on the *X*-axis were expressed in kg∙L^−1^. From the results of the partition coefficients determinations, the thermodynamics of solubilization by F127 and SBE-β-CD was disclosed by the excess free Gibbs energy ($\Delta G_{S}^{37{}^{\circ}C}$) evaluation, using the equation:

(12) $$\Delta G_{S}^{37{}^{\circ}C}=-RT\cdot \ln K_{excipient/buf}$$

### 2.11. Viscosity Measurements

The viscosity of F127 and SBE-β-CD solutions at concentrations used for the permeability experiments (1.0 *w*/*v*%, 2.0 *w*/*v*%, and 3.0 *w*/*v*%) was measured with the help of SV series Vibro Viscometer (A&D Company Ltd., Tokyo, Japan) with a high accuracy of 1% (standard deviation).

### 2.12. Measurements of the CBZ In Vitro Permeability

The apparent permeability coefficients were measured at 37 °C with the help of a vertical-type Franz diffusion cell (PermeGear, Inc., Hellertown, PA, USA). The experimental setup was the same as it was described in our previous study [40], where the excess amount of the investigated substance was placed in glass cups with ground-in lids. The required volume of buffer pH 6.8 without and with a predetermined concentration of the excipient (F127 or SBE-β-CD in the present study) was added, and the suspension was mixed overnight and filtered to obtain a clear solution. The concentration of this solution was determined and used for the further calculation of the permeability coefficient. The concentrations of the excipients were the same as used in the solubility experiments (1.33, 1.83, 2.30 mmol·L^−1^ and 6.89, 13.78, 20.67 mmol·L^−1^ of F127 and SBE-β-CD, respectively, which corresponded to 1%, 2%, and 3% (*w*/*v*)). Two kinds of artificial membranes were taken: a regenerated cellulose membrane MWCO 12–14 kDa (Standard Grade RC Dialysis Membrane, Flat Width 45 mm)—designated as RC, which was pretreated with water for 30 min and dried under air before the experiment—and the polydimethylsiloxane–polycarbonate (55% polydimethylsiloxane and 45% polycarbonate, 40 μm in thickness) membrane “Carbosil” (PENTAMED, Moscow, Russia, https://www.penta-med.ru (accessed on 2 November 2019)), abbreviated as PDS. The membrane was mounted between the donor and receptor chambers of the Franz cell. The effective surface area of the membrane was 0.785 cm^2^. Each experiment lasted 5 h. The donor solution was permanently mixing with a magnetic stirrer at a constant rate. The aliquots of the receptor solution (0.5 mL) were withdrawn every 30 min and promptly replaced with the same volume of the fresh buffer. The concentration of CBZ in the samples was determined by HPLC using the calibration curves. The flux of CBZ across the membrane (J) was derived from the cumulative amount of CBZ (Q) kinetic dependence normalized by the effective surface area of the membrane (A), according to the equation:

(13) $$J=\frac{dQ}{A\times dt}$$

The permeability coefficients were assessed under the sink conditions (at any experimental point the concentration of the compound in the receptor solution did not exceed 10% of the concentration in the donor solution) using the following equation:

(14) $$P_{app}=\frac{J}{C_{0}}$$

The apparent permeability coefficients were taken as the average values of no less than three replicas.

## 3. Results and Discussion

### 3.1. CMC and CAC Measurements

The surfactant molecules tend to self-associate forming aggregates in the form of micelles at a specific concentration called the critical micelle concentration (*CMC*). Unlike the surfactants, cyclodextrins has no *CMC* [39], but, as it has been reported [41], their molecules also self-associate to form aggregates above the critical aggregation concentration (*CAC*). Moreover, the fact that the aggregation behavior of cyclodextrin impacts the flux of the drug across the membrane was confirmed by Loftsson et al. [42]. All the premises provide convincing evidence of the feasibility of considering the *CMC* and *CAC* in the systems containing F127 and SBE-β-CD. The critical micelle concentration (*CMC*) of F127 and the critical aggregation concentration (*CAC*) of SBE-β-CD were determined by monitoring the refractive index by increasing the excipient concentration according to [34]. The method is based on the higher refractive index of micelles or aggregates when compared with monomers. The plots in Figure S1 illustrate the dependences of refractive indexes on the F127 or SBE-β-CD concentration in the absence and in the presence of CBZ, on which the point of intersection corresponds to the *CMC* of F127 and the *CAC* of SBE-β-CD. The values of the *CMC* and *CAC* are listed in Table 1.

Our value of the *CAC* of pure SBE-β-CD appeared to be the same as it was reported by Loftsson et al. [43]. As follows from Table 1, CBZ did not influence the *CAC* of cyclodextrin (the values were within the experimental error). Since F127 is widely used for the aim of the improvement of solubility in poorly soluble drugs, its capability of forming micelles in the solution has been investigated and discussed in many studies. Notably, the F127 *CMC* values reported in the literature are rather different. For example, *CMC* = 1.79 × 10^−4^ mol·L^−1^ (0.26 wt%), *CMC* = 5.55 × 10^−4^ mol·L^−1^, and *CMC* = 6.35 × 10^−4^ mol·L^−1^ were reported by Sharma et al. [44], Alexandridis et al. [45], and Leonties et al. [46] at 25 °C, respectively. Taking into account that the *CMC* of surfactant decreases with the accelerating temperature, our result seems to be in agreement with the literature data. The presence of CBZ was shown to decrease the *CMC* value of F127 from 1.640 × 10^−4^ mol·L^−1^ to 1.321 × 10^−4^ mol·L^−1^, which is in accordance with the results of Sharma et al. [44] for paclitaxel, tetracaine, methyl parabene, ethyl parabene, propyl parabene, and indomethacin.

### 3.2. Solubility Determinations

The equilibrium solubility of CBZ was determined in a phosphate buffer pH 6.8 and with the additions of F127 and SBE-β-CD (Table S1). The concentrations of the additives in the solubility experiments were 1.33, 1.83, 2.30 mmol·L^−1^ and 6.89, 13.78, 20.67 mmol·L^−1^ for F127 and SBE-β-CD, respectively, which corresponded to 1%, 2%, and 3% (*w*/*v*). The concentrations of the excipients (F127 and SBE-β-CD) were selected, taking into account that the most pronounced decrease in the permeability coefficient up to approximately 0.01–0.03 mol·L^−1^ (for cyclodextrins) and 0.003–0.005 mol·L^−1^ (for F127) is usually observed [11,13].

**Figure 2:**
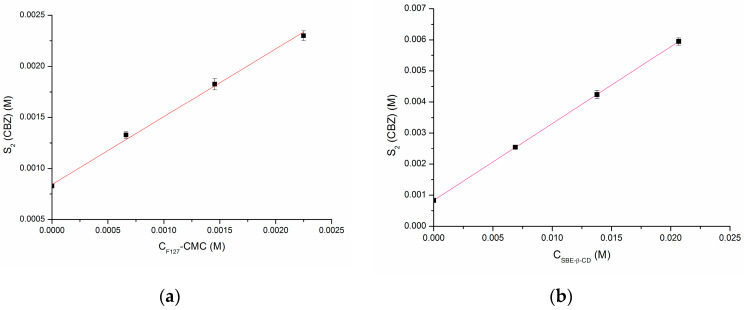
Phase solubility diagrams of CBZ at increasing F127 (**a**) and SBE-β-CD (**b**) concentrations; t = 37 °C. Data are shown as the mean ± SD of three or more observations.

The phase solubility diagrams are illustrated in Figure 2. The *CMC* value of F127 (Section 3.1) was used to calculate F127 micellar concentrations as the differences between the total *C*_*F*127_ and the *CMC*.

The solubility of CBZ measured in the range of the selected concentrations allowed us to determine the CBZ/F127 association constant and the CBZ/SBE-β-CD stability constant, which were then applied to estimate the free fraction of CBZ. The micellar association constant (*K_a_*) of CBZ with F127 and the apparent stability constant of the CBZ/SBE-β-CD complex (*K_c_*) were derived from the slope of the plots in Figure 2 using Equations (3) and (4). The values of *K_a_* = 2374.2 (±118.7) M^−1^ and *K_c_* = 395.9 (±15.8) M^−1^ were estimated for CBZ/F127 and CBZ/SBE-β-CD, respectively. In order to exclude the differences between the molecular weights of the excipients, the respective constants expressed in L∙kg^−1^ were derived and shown to be *K_a_* = 65.5 (±3.27) L∙kg^−1^ and *K_c_* = 247.5 (±5.23) L∙kg^−1^ which allows for the conclusion that these values agreed with the higher solubilization of CBZ by SBE-β-CD as compared to F127. Since CBZ is a thoroughly explored model drug, it seemed useful to compare our results with those reported before. The solubility of CBZ in 1.33 mmol·L^−1^ (1%) F127 solution at 37 °C was shown to be 1.43 × 10^−3^ mol·L^−1^ [47], which is in agreement with our results of 1.33 (±0.05)∙10^−3^ mol·L^−1^ (Table S1). The stability constants of CBZ with SBE-β-CD determined by Jain et al. [48] and Smith et al. [49] were 498.04 M^−1^ (unbuffered double distilled water, 30 °C) and 1035 M^−1^ (undefined pH and temperature). Taking into account the differences in the experimental conditions, our result agrees with that of Jain et al. [48].

Using the values of *K_a_* and *K_c_*, the free fraction of CBZ (*f_free_*) was determined at different F127 or SBE-β-CD concentrations by Equations (5) and (6) and listed in Table S2. The data in Table S2 demonstrated a more intensive reduction of the free CBZ concentration in the system with SBE-β-CD, when compared to F127 which agrees with a more pronounced increase in the CBZ apparent solubility in the presence of SBE-β-CD.

### 3.3. Solubilizing Capacity and Aggregation Behavior of F127 in the Presence of CBZ 

In order to reveal the impact of CBZ on the micelle-induced behavior of F127, the solubilizing potential of F127 towards CBZ was evaluated through the solubilizing capacity parameter (*χ*) (Equation (7)). The $\left(S_{2}(CBZ)-S_{2}^{0}(CBZ)\right)$ on $\left(C_{F-127}-CMC\right)$ dependence used for the *χ* calculation is illustrated in Figure S2. The value of the solubilizing capacity *χ* = 0.612 ± 0.009, which shows that 0.612 mole of CBZ is solubilized by one mole of F127. 

The aggregation number of F127 in the absence and in the presence of CBZ, which were calculated using Equations (8) and (9) and the Debye plots (Figure S3), were shown to be 85.7 ± 2.6 and 84.7 ± 3.1 without and with CBZ, respectively. This result clearly demonstrated that the presence of CBZ in the buffer solution of F127 does not influence the F127 aggregation behavior. It is worth noting that such parameters as aggregation numbers are extremely sensitive to the experimental conditions. In this connection, it is not surprising to observe rather different values of this parameter for F127 from various studies (from 3.7 [50] to 88.6 [51], and even 108 [52]). Such factors as experimental temperature, the pH of the solution, buffer composition, and even ambiguity in the molecular weights of different samples of the surfactant (the average molecular weight is indicated) can impact to the aggregation number. Despite the observed discrepancies in the aggregation numbers of F127, the experiments carried out in the same conditions with the same samples seem to be relevant within the study in order to make the comparative analysis of the main tendencies. The values determined in this study at 37 °C are in agreement with the study of Sharma et al. [51] for 25 °C, since the aggregation number is known to reduce with the temperature growth [53].

### 3.4. Determination of the CBZ Partition Coefficients F127 Micelles/Buffer and SBE-β-CD/Buffer

The micelle/buffer and cyclodextrin/buffer partition coefficients of drugs are useful to the evaluation of the water to the micelle phase and the water to the cyclodextrin partition processes. As it was reported in [38], the apparent micelle/buffer partition coefficient (*K_m/buf_*) is a ratio between the fraction of the compound (CBZ) in the micelle core pseudo phase and in the hydrophilic aqueous buffer phase (hydrated micelle corona plus aqueous solvent). In the case of cyclodextrins [39], the nonspecific interaction between the solute and the cavity of cyclodextrin takes place, and the solubilization process may be attributed to the partition of solutes from the aqueous buffer into the cyclodextrin cavity. In this work, the partition from the micellar pseudo phase (F127) to the aqueous buffer pH 6.8, as well as from SBE-β-CD to the buffer was studied using the plots of the linear dependences (Figure S4), plotted according to Equations (10) and (11). The calculations were conducted in two variants. In the first one, in which the concentration of the excipient was expressed in molarity, the values of the partition coefficients appeared to be *K_F127/buf_* = 737.63 (±10.90) and *K_SBE-β-CD/buf_* = 297.67 (±0.75) (Figure S4a,b). At first glance, the results look ambiguous since a greater solubility of CBZ with SBE-β-CD as compared to F127 was shown. To disclose the reason of this phenomenon, the dependences where the concentrations of the excipients on the X-axis were expressed in kg∙L^−1^ units were plotted and the partition coefficients values of *K_F127/buf_* = 58.54 (±0.86) and *K_SBE-β-CD/buf_* = 205.11 (±0.51) were derived (Figure S4c,d) in full agreement with the solubility results. It was interesting to compare the obtained partition coefficients with the well-established *logP*—the partition of CBZ from water to 1-octanol. The literature survey contains rather different values of *logP* for CBZ. The value of *logP* = 2.58 (*p* = 380.19) reported at pH 7.4 of the aqueous phase by [54], which fully coincides with that calculated by the program pDISOL-X [33], was taken in the present study for the sake of comparison. The conversion from the molar units to the mass units produced the value of *logP* (water→1-octanol) = 3.47. The comparative analysis demonstrated a higher tendency of CBZ to distribute in 1-octanol as compared to F127 and SBE-β-CD. The apparent free Gibbs energy of the CBZ transfer from the buffer to the polymer or cyclodextrin pseudo phases at the experimental temperature of 37 °C was evaluated using Equation (12). The results were obtained as follows: $\Delta G_{S}^{37{}^{\circ}C}$ (buffer pH 6.8→F127) = −10.5 kJ∙mol^−1^; $\Delta G_{S}^{37{}^{\circ}C}$ (buffer pH 6.8→SBE-β-CD) = −13.7 kJ∙mol^−1^ and were in agreement with the solubilization results.

### 3.5. Determination of the Membrane Permeability Coefficients

The permeability coefficients of CBZ in the pure buffer pH 6.8 and with the additions of F127 and SBE-β-CD were determined. The experimental donor solution concentrations and steady-state penetration fluxes are listed in Table S3. The values of the apparent permeability coefficients are placed in Table 2.

It should be emphasized that in order to ensure that the so-called aqueous boundary layer or unstirred water layer (UWL) did not affect the experimental permeability, we carried out a series of the experiments with and without agitation. The results demonstrated the independence of the permeability coefficient on stirring. Similar results have been reported by Dahan et al. [55,56] for progesterone and dexamethasone with PAMPA and Caco-2 cells, for carbamazepine in the PAMPA assay, and in the rat jejunal perfusion model [57]. In its turn, Brewster et al. [58] demonstrated the impact of UWL on the PAMPA permeability of carbamazepine. As it was estimated, for highly lipophilic compounds with CLOGP = 2.4 × 10^4^, diffusion across the UWL is a rate limiting step and should be taken into account [59]. The joint analysis of the literature data and our results leads to conclusion that the role of UWL is determined by several factors, such as the specificity of the membrane, experimental conditions, and properties of the drug. So, this role has to be estimated in each specific situation.

### 3.6. P_ratio_ Evaluation

**Figure 3:**
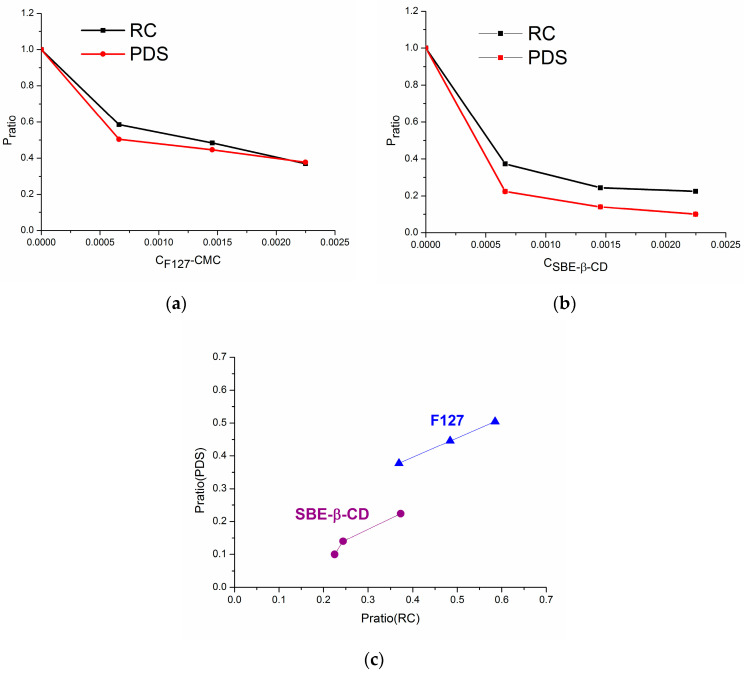
The effects of micellar concentration of F127 (**a**) and the concentration of SBE-β-CD (**b**) on CBZ permeation at 37 °C. The dependence between the *P_ratio_* values obtained with PDS on the *P_ratio_* values obtained with RC (**c**). *P_ratio_* values were calculated according to Equation (15).

Assessing the in vitro permeability for drugs intended for oral and percutaneous roots of administration is a precondition for the successful selection of a suitable dosage formulation, providing the maximal absorption and bioavailability [60]. An analysis of the permeability values determined for CBZ demonstrated an expected trend of lowering the permeability coefficients in the presence of the accelerating excipient concentration on the example of both membranes. The *P_ratio_* parameter was calculated in order to assess this trend quantitively [61]:

(15) $$P_{ratio}=\frac{P_{app}^{buf}}{P_{app}^{buf+excipient}}$$

where $P_{ratio}$ is a ratio of *P_app_* with the excipient ($P_{app}^{buf+excipient}$) to *P_app_* with pure buffer solutions ($P_{app}^{buf}$). The dependence of *P_ratio_* on the excipient concentration is illustrated in Figure 3.

The effect of the excipient is more pronounced for SBE-β-CD when compared to F127 (*P_ratio_*(SBE-β-CD) < *P_ratio_*(F127)) (Table 2, Figure 3a,b). An abrupt decrease in *P_ratio_* from zero to the minimal concentration of the excipient (more prominent in the system with SBE-β-CD) takes place. A further decrease demonstrated the convergence of the permeability coefficients across the RC and PDS membranes at the maximal concentration of F127 as an excipient. As opposed, in the presence of SBE-β-CD, the *P_ratio_* values for the RC membrane are higher than those for the PDS one. These results indicate both the membrane type and the nature of the excipient to be responsible for the permeability variations. Calculations of the CBZ/excipient association constants, as well as buffer/F127 and buffer/SBE-β-CD partition coefficients (Section 3.2 and Section 3.4), supported the permeability results since a greater affinity of CBZ to SBE-β-CD as compared to F127 was demonstrated. Figure 3c illustrates the dependence between *P_ratio_*(PDS) and *P_ratio_*(RC). Interestingly, as opposed to SBE-β-CD, a linear dependence with a high correlation coefficient and Fisher criterion between the *P_ratio_* for two membranes with F127 was derived and can be described by the equation:

*P_ratio_*(PDS) = 0.160(±0.004) + 0.588(±0.007)·*P_ratio_*(RC) *R* = 0.9999; *F* = 6336.62; *n* = 3 (16)

This result can be attributed to the similarity of the trends in the permeation across both membranes in the presence of F127. As opposed, the impact of SBE-β-CD on the permeation process seems to be more complicated and strongly dependent on the membrane. It might come from the negative charge on SBE-β-CD which can provoke the membrane surface modifications as a consequence of the SBE-β-CD-PDS membrane interactions, resulting in retarding the diffusion of CBZ across the membrane [62]. In this case, at least two phenomena determine the permeability—the interaction between CBZ and SBE-β-CD and between SBE-β-CD and the membrane, as opposed to CBZ with a non-ionic surfactant, F127.

### 3.7. Disclosing the Permeability Variations Using a Quasi-Equilibrium Transport Model

At the next step, to the aim of quantitatively disclosing the permeability variations, we used a quasi-equilibrium mathematical mass transport model developed to describe the solubility–permeability interplay proposed in the studies of Dahan, Miller, and co-workers [11,12] for the solubility-enabling formulations containing cyclodextrins, cosolvents, etc. Considering the concept based on the existence of the solubility–permeability interplay in the presence of different solubilizing agents, the apparent membrane permeability ($P_{app}^{calc}$) in the presence of a specific excipient concentration can be calculated using the drug apparent solubility at a specific excipient concentration ($S_{2}(CBZ)$), the solubility in pure buffer ($S_{2}^{0}(CBZ)$), and the apparent permeability in pure buffer ($P_{app}^{0}$) [11,12], such as:

(17) $$P_{app}^{calc}=\frac{P_{app}^{0}\cdot S_{2}^{0}(CBZ)}{S_{2}(CBZ)}$$

**Figure 4:**
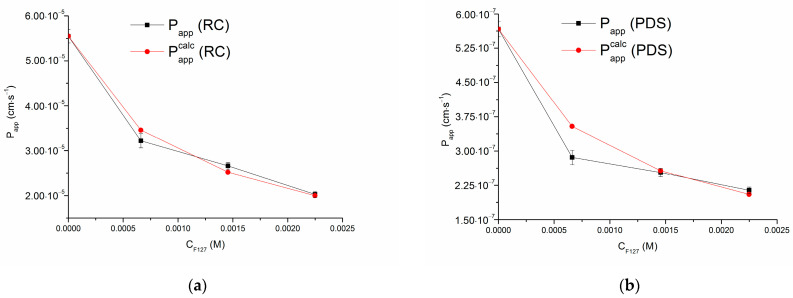

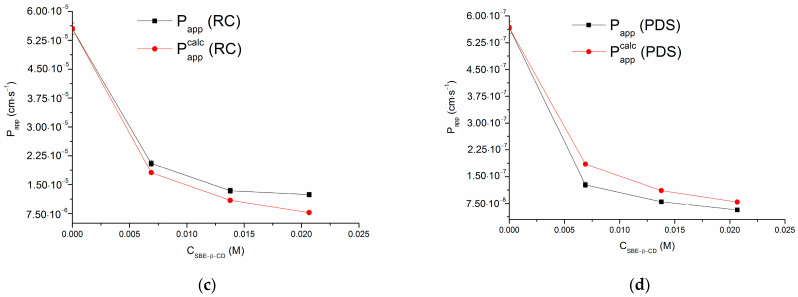
Calculated ($P_{app}^{calc}$) and experimental ($P_{app}$) apparent permeability coefficients of CBZ across RC with F127 (**a**), PDS with F127 (**b**), RC with SBE-β-CD (**c**), PDS with SBE-β-CD (**d**) at increasing F127 and SBE-β-CD concentrations; t = 37 °C.

The values of $P_{app}^{calc}$ are listed in Table 2. Figure 4 illustrates the comparison between the experimental and calculated apparent permeability of CBZ across RC and PDS with increasing F127 and SBE-β-CD concentrations.

According to the plots in Figure 4, better agreement between $P_{app}$ and $P_{app}^{calc}$ was achieved for the CBZ permeation in the presence of F127 micelles (especially across the RC membrane). In turn, with SBE-β-CD in the donor solution, the $P_{app}^{calc}$ values are somewhat lower with RC and higher with PDS membranes than the experimental $P_{app}$ values. These findings allow the proposal of additional mechanisms that influence permeability, going beyond the model approach which primarily relied on solubility for quantitatively predicting permeability. The underestimation of the permeability through the PDS membrane with SBE-β-CD can probably be attributed to the specific interaction of anionic cyclodextrin with PDS, as it was underlined above. In addition, as it was reported in [57], decreased diffusivity can be attributed to the increased viscosity of the donor solution upon the acceleration of the excipient concentration. The solvation of CBZ by the solvent molecules increases in the presence of the excipients, which results in the increasing of the hydrodynamic radius which, in turn, hampers the diffusion in the bulk donor solution [14]. Fischer et al. [63] proposed that just a change in the viscosity of the aqueous donor compartment might be the reason of the decreased permeability of carboxyfluorescein in the non-ionic surfactant poloxamer 188. To support the obtained results, the dynamic viscosity (MPa) of the dissolution medium was measured at the experimental temperature of 37 °C and listed in Table S4. As expected, the viscosity was increased both with F127 and SBE-β-CD in solution. However, even tentative calculations clearly showed that such small changes in viscosity cannot be responsible for the differences between the experimental and calculated permeability coefficients.

### 3.8. Correction of Permeability Coefficients for the Free Drug Concentration

The authors [16] underlined that when different excipients, such as cyclodextrins or micelle-forming polymers, are used, it is necessary to obtain “true” permeability values by a correction of permeability coefficients for the free drug concentration. In this study, the free drug concentration in the presence of F127 and SBE-β-CD was assessed indirectly through the equilibrium solubility experiments at all the excipient concentrations (Table 2). The values of the experimental apparent permeability coefficients (*P_app_*) were recalculated, taking into account the free CBZ concentration in the donor solutions ($C_{0}^{free}$) in order to obtain the absolute permeability coefficients values ($P_{abs}$) (Table 2). The concentrations of the free CBZ in the donor solutions ($C_{0}^{free}$) and the absolute permeability coefficients ($P_{abs}$) [18] were calculated using the values of f_free_ using the following equations:

(18) $$C_{0}^{free}=C_{0}\cdot f_{free}$$

(19) $$P_{abs}=\frac{J}{C_{0}^{free}}$$

The values of $P_{abs}$ approved the rule reported in [18] that $P_{app}$ is controlled by the free drug fraction. Following from the very low drug/CD binding constant (*Kc* = 395.9 M^−1^), a rapid exchange of the CBZ/CD complex to free CBZ is possible. In order to reveal the role of the free fraction on the permeability and to understand the influence of other factors on the permeation in the presence of the excipients, we calculated the difference between the permeability coefficients in the pure buffer and the corrected values as $\Delta P=(P_{app}-P_{abs})$ (Table 2). It seems reasonable to believe that if ΔP is positive ($P_{app}$ > $P_{abs}$), then some possibility of micellar or complexed drug partition to the membrane can be proposed. In the opposite case ($P_{app}$ ˂ $P_{abs}$), the impact of additional decreasing factors on the transition through the membrane is reliable [64]. The analysis of ΔP from Table 2 led to the following conclusions. Both the nature of the excipient and the membrane properties are significant for the CBZ permeation mode. The ΔP parameter for the case of RC and F127 as an excipient takes on a small positive value at the minimal F127 concentration, implying only negligible possibilities of the micellar drug permeation. The further increase in the F127 concentration results in the negative ΔP values very close to zero, meaning that the concentration of the drug free in the solution can be the only driving force for membrane transport. Contrarily, increasing with the SBE-β-CD concentration (especially at the maximal value) the negative ΔP (with the same RC membrane) clearly indicates a growing impact of some other (apart from free drug concentration) factors on the CBZ permeation. First of all, let us address to the SBE-β-CD capability of self-association forming aggregates in aqueous solutions (Section 3.1). As it was underlined, the cyclodextrin aggregates can impact the membrane transport of the drug. The critical aggregation concentration of SBE-β-CD − *CAC* = 0.017 mol·L^−1^ was determined in this study, which is less than the maximal *C_SBE-β-CD_* = 0.021 mol·L^−1^, implying CD aggregates in the solution, which most probably hamper the permeation rate and can be the reason of a dramatic growth of the ΔP negativity. Moreover, possible interactions between the hydrophilic membrane and the hydrophilic SBE-β-CD increasing with the CD concentration growth are also responsible for ΔP ˂ 0. Interestingly, the case of PDS ΔP > 0 and only slightly varies from 1.01 to 0.91 upon the SBE-β-CD concentration growth. Evidently, in the case of SBE-β-CD, the nature of the membrane is a crucial factor for CBZ permeability. In turn, the trends of ΔP variations in the presence of F127 in the solution are very close between the RC and PDS membranes.

## 4. Conclusions

The findings of this study demonstrated a clear effect of pharmaceutical excipients, such as a non-ionic surfactant F127 and anionic SBE-β-CD on the permeation of CBZ. An inverse relationship between the solubility and permeability of CBZ in the solutions of F127 and SBE-β-CD was revealed. A greater affinity of CBZ to SBE-β-CD, as compared to F127, was demonstrated by the calculations of the CBZ/excipient association constants, as well as buffer/F127 and buffer/SBE-β-CD partition coefficients. The effect of the excipient on the solubility and permeability of CBZ was also shown to be more pronounced for SBE-β-CD as compared to F127. The influence of SBE-β-CD on the CBZ permeation process was more complicated and membrane dependent as compared to F127. The results indicated that additional mechanisms to the permeability, apart from the model approach within the quasi-equilibrium mathematical mass transport model, enabled the quantitative prediction of the permeability as a function of the solubility. The correction of the permeability coefficients for the free CBZ concentration approved that, in the case of SBE-β-CD, the nature of the membrane is a crucial factor for CBZ permeability. In turn, the trends of permeability in the presence of F127 in the solution are very close for the RC and PDS membranes. The present study provides an additional insight into CBZ permeability in the presence of solubilizing excipients and discloses the permeability tendencies from the point of the excipient properties and membrane nature. The findings of the present work points once again to the notion that the free drug fraction determination is a key parameter for permeability and, possibly, for the prediction of in vivo absorption.

## 5. Future Perspectives

From the results of the present study, it seems that there is still a lot of room for study within the scope of the solubility–permeability relationship in the complicated systems. 

Further future work will establish (1) if the presented approach is applicable to other solubility enhancing additives, for example, hydrotropic agents, co-solvents, and polymers that are not capable of forming the micelles; (2) how does this approach work on ionizable compounds at different pH values of the medium?; (3) if this approach is applicable to multicomponent systems containing several solubilizing agents of different nature, including those joining cyclodextrins and ions (implying hydroxyl acids and other organic acids and bases, for example, sodium acetate, sodium benzoate, benzalkonium chloride).

## Figures and Tables

**Table 1:** Critical micelle concentrations (*CMC*) and critical aggregation concentrations (*CAC*), determined at 37 °C.

Conditions	*CMC* (F127) (mol·L^−1^)	*CAC* (SBE-β-CD) (mol·L^−1^)
in the absence of CBZ	1.640 (±0.066)∙10^−4^	1.716 (±0.069)∙10^−2^
*C_CBZ_* = 4.06∙10^−4^ mol·L^−1^	1.321 (±0.053)∙10^−4^	1.724 (±0.069)∙10^−2^

**Table 2:** Experimental values of the apparent permeability coefficients (*P_app_*), a ratio of *P_app_* with the excipient to *P_app_* with pure buffer solution (*P_ratio_*), calculated value of the permeability coefficient ($P_{app}^{calc}$), free CBZ concentration in the donor solutions ($C_{0}^{free}$), absolute permeability coefficients ($P_{abs}$), and ΔP at 37 °C.

C_exipient_ (mmol·L^−1^)			Permeability Results			
	*P_app_* (cm∙s^−1^)	$P_{ratio}$ ^1^	$P_{app}^{calc}$ (cm·s^−1^) ^2^	$C_{0}^{free}$ (mol·L^−1^) ^3^	$P_{abs}$ (cm·s^−1^) ^4^	ΔP ^5^
	RC					
0	(5.55 ± 0.15) × 10^−5^	-	-	-	(5.55 ± 0.15) × 10^−5^	-
	F127					
1.33	(3.22 ± 0.16) × 10^−5^	0.585	3.46 × 10^−5^	4.73 × 10^−4^	4.99 × 10^−5^	0.56 × 10^−5^
1.83	(2.66 ± 0.08) × 10^−5^	0.484	2.52 × 10^−5^	3.77 × 10^−4^	5.86 × 10^−5^	−0.31 × 10^−5^
2.30	(2.03 ± 0.07) × 10^−5^	0.369	2.00 × 10^−5^	4.73 × 10^−4^	5.82 × 10^−5^	−0.27 × 10^−5^
	SBE-β-CD					
6.89	(2.05 ± 0.08) × 10^−5^	0.373	1.81 × 10^−5^	4.46 × 10^−4^	7.15 × 10^−5^	−1.6 × 10^−5^
13.78	(1.34 ± 0.07) × 10^−5^	0.244	1.09 × 10^−5^	3.88 × 10^−4^	7.96 × 10^−5^	−2.41 × 10^−5^
20.67	(1.24 ± 0.06) × 10^−5^	0.225	7.74 × 10^−6^	5.59 × 10^−4^	1.05 × 10^−4^	−4.95 × 10^−5^
	PDS					
0	(5.67 ± 0.17) × 10^−7^	-	-	-	(5.67 ± 0.17) × 10^−7^	
	F127					
1.33	(2.86 ± 0.16) × 10^−7^	0.504	3.54 × 10^−7^	4.64 × 10^−4^	4.41 × 10^−7^	1.26 × 10^−7^
1.83	(2.53 ± 0.09) × 10^−7^	0.446	2.57 × 10^−7^	3.78 × 10^−4^	5.56 × 10^−7^	0.11 × 10^−7^
2.30	(2.14 ±0.07) × 10^−7^	0.377	2.05 × 10^−7^	3.12 × 10^−4^	6.12 × 10^−7^	−0.45 × 10^−7^
	SBE-β-CD					
6.89	(1.27 ± 0.08) × 10^−7^	0.224	1.85 × 10^−7^	4.40 × 10^−4^	4.66 × 10^−7^	1.01 × 10^−7^
13.78	(7.96 ± 0.20) × 10^−8^	0.140	1.11 × 10^−7^	5.14 × 10^−4^	4.72 × 10^−7^	0.95 × 10^−7^
20.67	(5.65 ± 0.15) × 10^−8^	0.100	7.91 × 10^−8^	5.00 × 10^−4^	4.76 × 10^−7^	0.91 × 10^−7^

^1^ $P_{ratio}=\frac{P_{app}^{buf}}{P_{app}^{buf+excipient}}$; ^2^ $P_{app}^{calc}=\frac{P_{app}^{0}\cdot S_{2}^{0}(CBZ)}{S_{2}(CBZ)}$; ^3^ $C_{0}^{free}=C_{0}\cdot f_{free}$; ^4^ $P_{abs}=\frac{J}{C_{0}^{free}}$; ^5^ $\Delta P=(P_{app}-P_{abs})$.

## Data Availability

The results obtained for all experiments performed are shown in the manuscript and Supplementary Materials, the raw data will be provided upon request.

## Supplementary Materials

The following supporting information can be downloaded at: https://www.mdpi.com/article/10.3390/pharmaceutics16020184/s1, Figure S1: Refractive index vs. concentration of F127 pure (a), F127 in the presence of CBZ (b), SBE-β-CD pure (c), and SBE-β-CD in the presence of CBZ (d) at 37 °C; Figure S2: Plot of $\left(S_{2}(CBZ)-S_{2}^{0}(CBZ)\right)$ on $\left(C_{F-127}-CMC\right)$ dependence at different concentrations of F127 in buffer pH 6.8, 37 °C; Figure S3: Debye-plots for systems of F127 (black squares) and F127+CBZ (red circles) in buffer pH 6.8; Figure S4: Plots of $\left(S_{2}(CBZ)-S_{2}^{0}(CBZ))/S_{2}^{0}(CBZ\right)$ on (*C*_*F*127_ − *CMC*) (a,c) and *C_SBE-β-CD_* (b,d) dependences used for the F127/buffer and SBE-β-CD/buffer partition coefficients calculation (a) and (b)—from molar concentrations, (c) and (d)—from the concentrations expressed in kg∙L^−1^ units; 37 °C; Table S1: Solubility of CBZ at 37.0 ± 0.1 °C; Table S2: Free fraction (*f_free_*) of CBZ at different excipient concentrations; Table S3: Donor solution concentrations (*C*_0_) and steady state flux (J) of CBZ in pure buffer pH 6.8 and in F127 and SBE-β-CD solutions at 37 °C; Table S4: Viscosity of the donor solutions at 37 °C.

## Abbreviations

*CMC*	Critical micelle concentration
*CAC*	Critical aggregation concentration
*K_a_*	Association constant
*K_c_*	Stability constant
*C_total_*	Total CBZ concentration
*C_free_*	Free drug concentration
*C_micellar_*	Micellar (F127) drug concentration
*C_complex_*	Drug concentration in the CBZ/SBE-β-CD complex.
*C*_*F*127_	Concentration of F127
*C_SBE-β-CD_*	Concentration of SBE-β-CD
*f_free_*	fraction of free CBZ molecules
$S_{2}^{0}(CBZ)$	Equilibrium CBZ solubility in the saturated solution of pure buffer
$S_{2}(CBZ)$	CBZ equilibrium solubility in the presence of F127 or SBE-β-CD
*χ*	Solubilizing capacity
*N_agg_*	Aggregation number
*M_w_* (*micelle*)	Molecular weight of F127 micelle
*M_w_* (*monomer*)	Molecular weight of F127 monomer
*K*_*m*/*buf*_ = *K*_*F*127/*buf*_	Micelle (F127)/buffer partition coefficient
*K*_*SBE*-*β*-*CD*/*buf*_	SBE-β-CD/buffer partition coefficient
*RC*	Regenerated cellulose membrane
*PDS*	Polydimethylsiloxane-polycarbonate membrane
*J*	Steady state flux through the membrane (µmol∙cm^−2^∙s^−1^)
*P_app_*	Permeability coefficient (cm∙s^−1^)
*P_ratio_*	A ratio of P_app_ with the excipient to P_app_ with pure buffer solutions
$P_{app}^{calc}$	Permeability coefficient calculated within a quasi-equilibrium mathematical mass transport model
$P_{abs}$	Absolute permeability coefficient for the free drug concentration
ΔP	Difference between the permeability coefficients in pure buffer and the corrected values
