# Supplementary material for: Mechanistic Insight in Permeability through Different Membranes in the Presence of Pharmaceutical Excipients: A Case of Model Hydrophobic Carbamazepine
Source: Pharmaceutics. 2024 Jan 28;16(2):184. doi: 10.3390/pharmaceutics16020184 (PMC10893053; doi:10.3390/pharmaceutics16020184)
Supplement: Supplementary file 1 [file pharmaceutics-16-00184-s001.zip › pharmaceutics-2799750-supplementary.pdf]

Supplementary Material

Table of Contents

Figure S1	Refractive index vs. concentration of F127 pure (a), F127 in the presence of CBZ (b), SBE-β-CD pure (c), and SBE-β-CD in the presence of CBZ (d) at 37 °C.
Figure S2	Plot of $(S_2(CBZ) - S_2^0(CBZ))$ on $(C_{F-127} - CMC)$ dependence at different concentrations of F-127 in buffer pH 6.8, 37 °C.
Figure S3	Debye-plots for systems of F-127 (black squares) and F127+CBZ (red circles) in buffer pH 6.8.
Figure S4	Plots of $(S_2(CBZ) - S_2^0(CBZ))/S_2^0(CBZ)$ on (C_{F127}-CMC) (a,c) and $C_{SBE\text{-}\beta\text{-}CD}$ (b,d) dependences used for the F127/buffer and SBE-β-CD/buffer partition coefficients calculation (a) and (b) - from molar concentrations, (c) and (d) - from the concentrations expressed in kg·L^{-1} units; 37 °C.
Table S1	Solubility of CBZ at 37.0±0.1°C.
Table S2	Free fraction (f_{free}) of CBZ at different excipient concentrations.
Table S3	Donor solution concentrations (C_0) and steady state flux (J) of CBZ in pure buffer pH 6.8 and in F127 and SBE-β-CD solutions at 37 °C.
Table S4	Viscosity of the donor solutions at 37 °C.

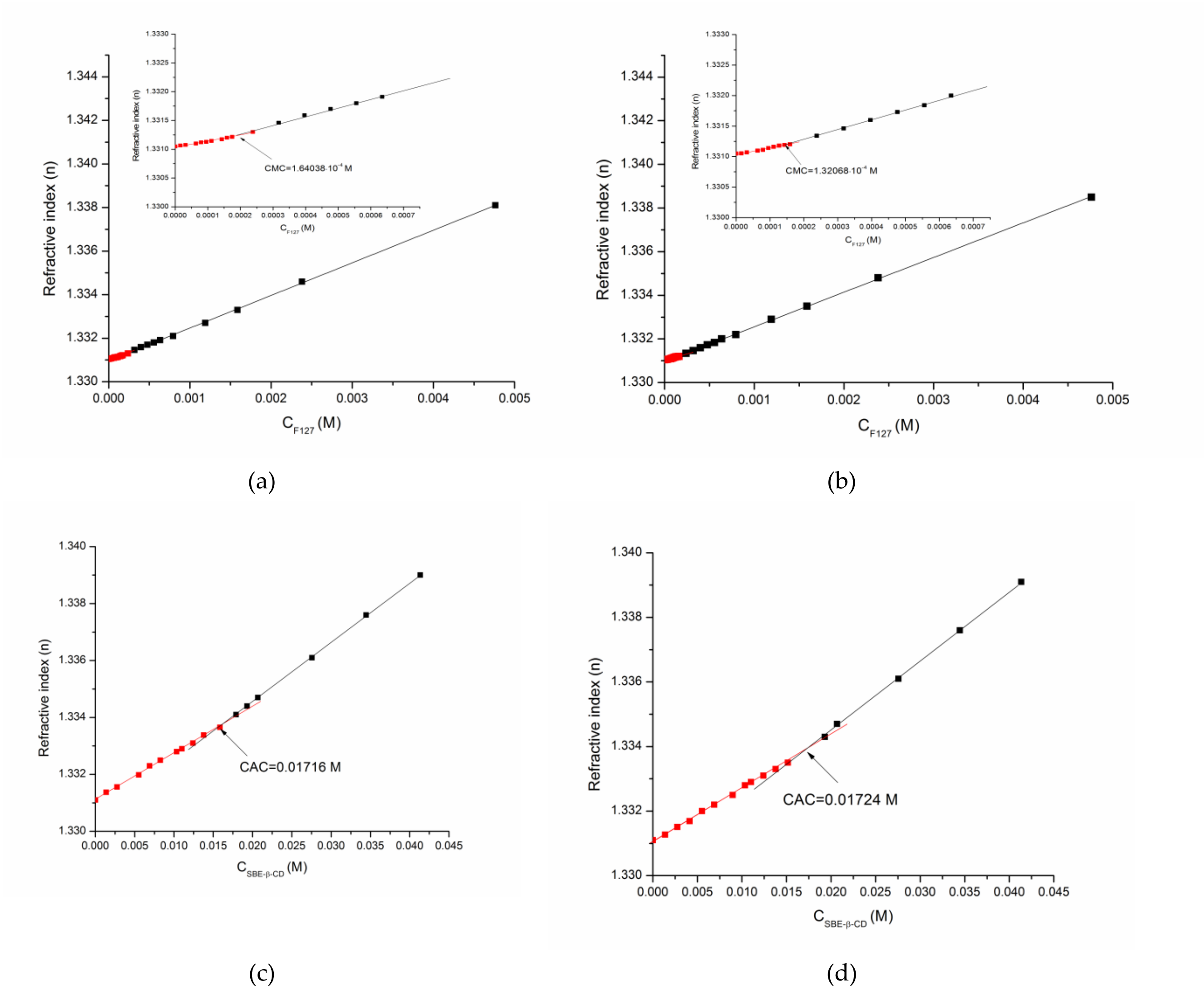

Figure S1. Refractive index vs. concentration of F127 pure (a), F127 in the presence of CBZ (b), SBE-β-CD pure (c), and SBE-β-CD in the presence of CBZ (d) at 37 °C.

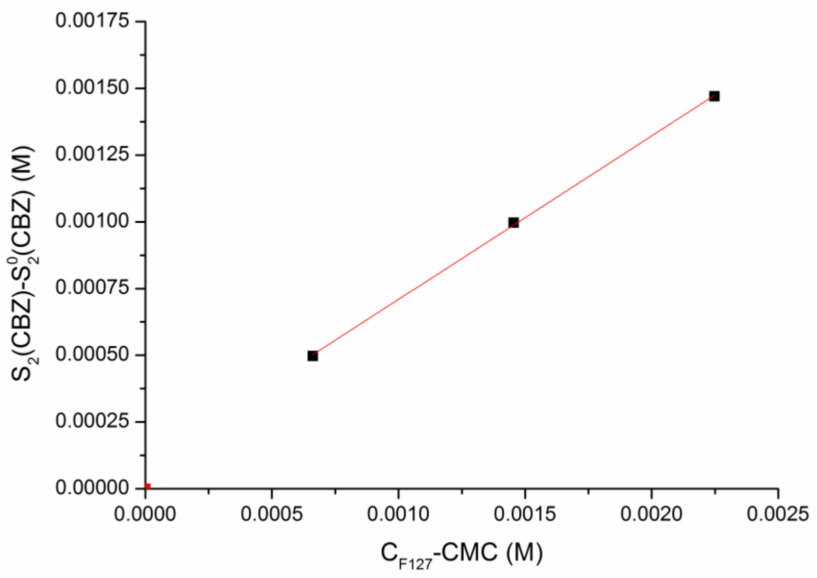

Figure S2. Plot of $(S_2(CBZ)-S_2^0(CBZ))$ on $(C_{F-127}-CMC)$ dependence at different concentrations of F-127 in buffer pH 6.8, 37 °C.

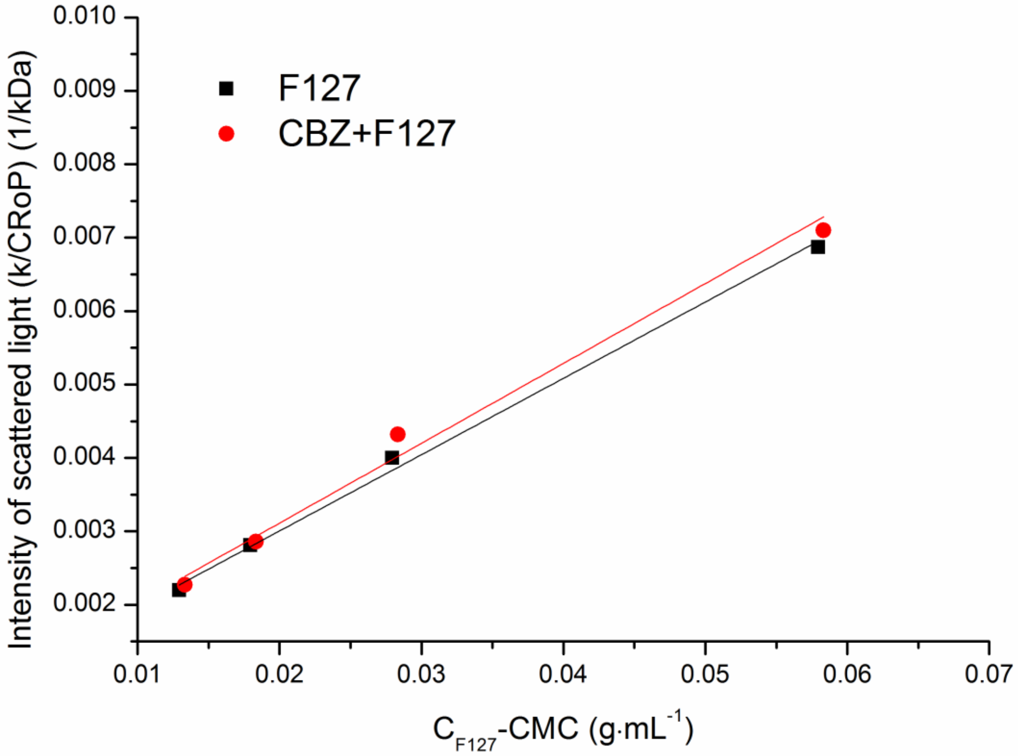

Figure S3. Debye-plots for systems of F-127 (black squares) and F127+CBZ (red circles) in buffer pH 6.8.

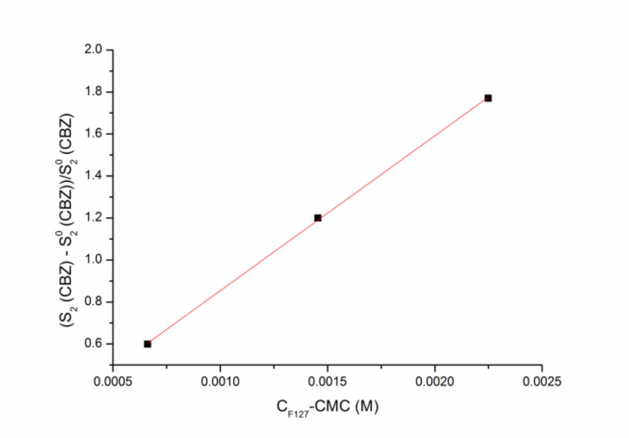

(a)

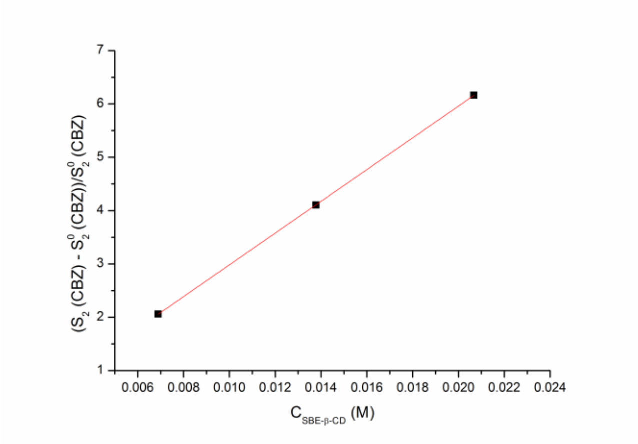

(b)

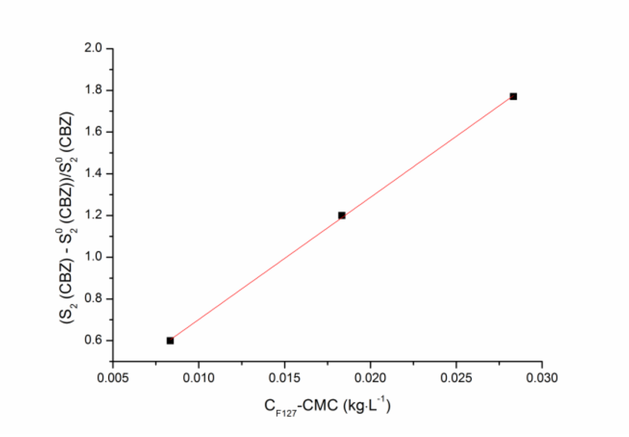

(c)

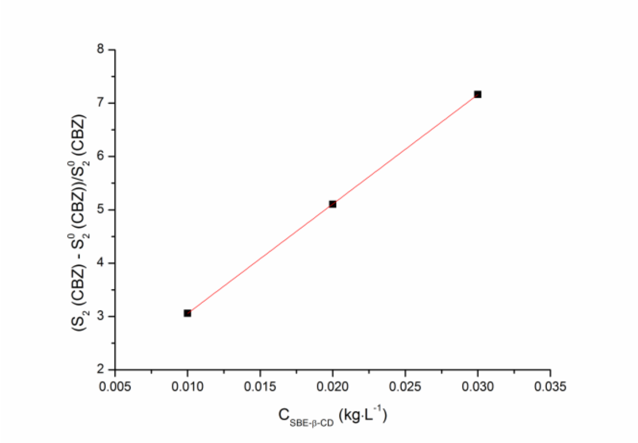

(d)

Figure S4. Plots of $(S_2(CBZ) - S_2^0(CBZ)) / S_2^0(CBZ)$ on (C_{F127}-CMC) (a,c) and $C_{SBE\text{-}\beta\text{-}CD}$ (b,d) dependences used for the F127/buffer and SBE-β-CD/buffer partition coefficients calculation (a) and (b) - from molar concentrations, (c) and (d) - from the concentrations expressed in kg·L^{-1} units; 37 °C.

Table S1. Solubility of CBZ at 37.0±0.1ºC.

F-127concentration (mmol·L^{-1})	Solubility in buffer pH 6.8+F-127 (mol·L^{-1})	SBE-β-CD concentration (mmol·L^{-1})	Solubility in buffer pH 6.8+SBE-β-CD (mol·L^{-1})
0	(8.30±0.14)·10^{-4}	0	(8.30±0.14)·10^{-4}
1.33	(1.33±0.05)·10^{-3}	6.89	(2.54±0.06)·10^{-3}
1.83	(1.83±0.06)·10^{-3}	13.78	(4.24±0.11)·10^{-3}
2.30	(2.30±0.08)·10^{-3}	20.67	(5.95±0.14)·10^{-3}

Table S2. Free fraction (f_{free}) of CBZ at different excipient concentrations.

F-127concentration (mmol·L^{-1})	Free fraction of CBZ	SBE-β-CD concentration (mmol·L^{-1})	Free fraction of CBZ
0	1	0	1
1.33	0.647	6.89	0.288
1.83	0.454	13.78	0.168
2.30	0.350	20.67	0.119

Table S3. Donor solution concentrations (C_0) and steady state flux (J) of CBZ in pure buffer pH 6.8 and in F127 and SBE-β-CD solutions at 37 ºC.

$C_{exipient}$	Initial parameters of the permeation experiments	
(mmol·L^{-1})	C_0 (mol·L^{-1})	J (μM·cm^{-2}·s^{-1})
	RC	
0	5.95·10^{-4}	3.31·10^{-5}
	F127	
1.33	7.31·10^{-4}	2.36·10^{-5}
1.83	8.31 ·10^{-4}	2.21·10^{-5}
2.30	1.350·10^{-3}	2.75·10^{-5}
	SBE-β-CD	
6.89	1.55·10^{-3}	3.19·10^{-5}
13.78	2.31·10^{-3}	3.09·10^{-5}
20.67	4.71·10^{-3}	5.85·10^{-5}
	PDS	
0	4.97·10^{-4}	2.82·10^{-7}
	F127	
1.33	7.18·10^{-4}	2.05·10^{-7}
1.83	8.31·10^{-4}	2.10·10^{-7}
2.30	8.92·10^{-4}	1.91·10^{-7}
	SBE-β-CD	
6.89	1.53·10^{-3}	1.94·10^{-7}
13.78	3.06·10^{-3}	2.43·10^{-7}
20.67	4.21·10^{-3}	2.38·10^{-7}

Table S4. Viscosity of the donor solutions at 37 ºC.

F-127concentration (mmol·L^{-1})	Viscosity (MPa)	SBE-β-CD concentration (mmol·L^{-1})	Viscosity (MPa)
0	0.71	0	0.71
1.33	0.84	6.89	0.72
1.83	0.88	13.78	0.75
2.30	1.09	20.67	0.80
